# RNA binding properties of the US11 protein from four primate simplexviruses

**DOI:** 10.1186/1743-422X-8-504

**Published:** 2011-11-03

**Authors:** Sarah Tohme, Cyprian D Cukier, Alberto Severini

**Affiliations:** 1 National Microbiology Laboratory, Public Health Agency of Canada, 1015 Arlington Street, Winnipeg, MB, R3E 3R2, Canada; 2 Dept. of Medical Microbiology and Infectious Diseases, University of Manitoba, 745 Bannatyne Avenue, Winnipeg, Manitoba R3E 0J9, Canada; 3 Department of Medical Biochemistry and Biophysics, Karolinska Institutet, SE-171 77 Stockholm, Sweden

## Abstract

**Background:**

The protein encoded by the Us11 gene of herpes simplex viruses is a dsRNA binding protein which inhibits protein kinase R activity, thereby preventing the interferon-induced shut down of protein synthesis following viral infection. Us11 protein is not essential for infectivity *in vitro *and in mice in herpes simplex virus type 1 (HSV1), however this virus has a second, and apparently more important, inhibitor of PKR activity, the γ_1_34.5 protein. Recently sequenced simian simplexviruses SA8, HVP2 and B virus do not have an ORF corresponding to the γ_1_34.5 protein, yet they have similar, or greater, infectivity as HSV1 and HSV2.

**Methods:**

We have expressed the US11 proteins of the simplexviruses HSV1, HSV2, HVP2 and B virus and measured their abilities to bind dsRNA, in order to investigate possible differences that could complement the absence of the γ_1_34.5 protein. We employed a filter binding technique that allows binding of the Us11 protein under condition of excess dsRNA substrate and therefore a measurement of the true Kd value of Us11-dsRNA binding.

**Results and Conclusions:**

The results show a Kd of binding in the range of 0.89 nM to 1.82 nM, with no significant difference among the four Us11 proteins.

## Introduction

The genus Simplexvirus comprises a number of closely related herpesviruses with very similar genetic structure and life cycles. Most simplexviruses, like the human simplex viruses types 1 and 2 (HSV 1 and HSV 2), B virus (*Cercopithecine herpesvirus 1)*, SA8 (*Cercopithecine herpesvirus 2) *and herpes virus papio type 2 (*Cercopithecine herpesvirus *16), infect primates and produce very similar oro-genital lesions in their natural hosts and may cause encephalitis or severe infections in mice and other hosts [1]. For example, B virus causes mostly oro-genital lesions in macaques, but if transmitted to humans produces an often fatal ascending encephalomyelitis [1,2].

Complete sequencing of the genomes of simian simplexviruses have confirmed the close genetic homology [3-5]. All the genes are conserved and collinear, except for the γ_1_34.5 gene (RL1) which is present in HSV-1 and HSV-2, but absent from the simian simplexviruses B virus, SA8 and HVP2.

The γ_1_34.5 protein is an important virulence factor in HSV-1. Null mutants for γ_1_34.5 loose their virulence in mice and they show a severely impaired replication in cells culture [6,7]. These mutants can be rescued by a compensatory mutation that puts the US11 ORF under the control of an immediate early promoter [8-10]. In fact, both γ_1_34.5 and US11 proteins act by inhibiting the activation of the interferon-dependent protein kinase R (PKR) response. PKR is activated by binding to dsRNA and it phosphorylates the ribosomal translation factor eIF2α, thereby blocking cellular protein synthesis. γ_1_34.5 promotes de-phosphorylation of eIF2α [7,11] while US11 inhibits PKR activation by binding to dsRNA [11,12]. Since simian simplexviruses lack the γ_1_34.5 gene and yet replicate very well in culture and may show neurovirulence in mice, it is tempting to speculate that US11 may take over the entire inhibition of the PKR system in these viruses.

US11 is a ribosome associated protein which is produced late in infection and is packaged into the viral tegument at approximately 1000 copies [13,14]. US11 is not thought to be necessary for early gene expression or DNA replication, however it has been found that HSV 1 relies more heavily on US11 in later stages of its lifecycle to prevent an antiviral state [15-17].

US11 is thought to inhibit PKR via the sequestering of dsRNA [11]. US11 has also been shown to inhibit PKR activation by binding to PACT, an RNA-independent mechanism of activating PKR [18]. In addition, US11 has recently been shown to counteract OAS (2'-5' oligoadenylate synthetase), an interferon inducible gene also activated by dsRNA, in part by sequestering dsRNA through its C-terminal domain [19].

US11 binds dsRNA via a unique motif comprised of a set of R-X-P repeats at the C-terminal end of the gene. The R-X-P motifs bind to dsRNA with high affinity, with Kds reported in the range of 12 to 70 nM [12,20]. US11 bind to dsRNA of minimum length of 39 bp and preferentially binds RNA structures of higher-order and greater sequence complexity [12,20].

Within the simplexviruses that express US11, the protein is highly conserved except for the number of R-X-P at the C-terminal end of the protein. HSV 1 contains 20 to 24 copies of R-X-P repeats, the number of repeats has been shown to vary among viral strains but is not a strain specific feature. HSV2 contains 19-20 repeats and this also is thought to vary slightly among strains. Within the non-human primate viruses; B virus (strain E2490) contains 20 R-X-P repeats, SA8 strain B264 contains only 10 while HVP2 (strain X313) has 32 R-X-P repeats, although there is considerable variation in the number of repeats among HVP2 strains.

In this work we have expressed in E. coli US11 proteins from the human simplexvirus HSV-1, HSV-2, and the simian simplexviruses HVP-2 and B-virus and we investigated if there are differences in dsRNA binding activities that could indicate different roles of US11 in the two groups of simplexviruses.

## Materials and methods

### Cell Culture and Viruses

Vero cells (ATCC number CCL-81) were maintained in Dulbecco's modified Eagle's medium (DMEM) supplemented with 5% calf serum. HSV 1 (strain F), HSV 2 (strain G) were obtained from The American Tissue Culture Collection (ATCC numbers VR-733 and VR-734, respectively). Herpes papio 2 (HVP2, strain X313), originally isolated in Dr. Richard Eberle's laboratory, University of Oklahoma, and B virus (strain E2490) were obtained from the Southwest Foundation for Biomedical Research, San Antonio, TX, USA. Simian agent 8 (SA8, strain B264) was donated by Dr. Heinz Feldman, National Microbiology Laboratory, Winnipeg Manitoba. Viral genomic DNA was prepared as previously described [4]. Genomic DNA from B virus was prepared in the biosafety level 4 facility at the National Microbiology Laboratory of the Public Health Agency of Canada.

### US11 Recombinant Protein Expression and Purification

The US11 gene was amplified by polymerase chain reaction (PCR) using the following sets of primers: HSV1, forward 5'GGAATTCCATATGAGCCAGACCCAACC3' and reverse 5'CCCAAGCTTCTATACAGACCCGCGAG3'; HSV2, forward 5' GGAATTCCATATGGCATCCGGGGTT3' and reverse 5'CCCAAGCTTCTAGGCAAGCCCGCG3'; B virus, forward 5'GGAATTCCATATGCTAATGGCGTCAA3' and reverse 5'CCCAAGCTTGAAACCTCATCAACCC3'; SA8, forward 5'GGAATTCCATATGGCGTCCGCGCCC3' and reverse 5'CCCAAGCTTAGGGGGCCGTCCTCACC3'; and HVP2 forward 5'GGAATTCCATATGGCGTCCGTGGCC3' and reverse 5'CCCAAGCTTAGGGGGCCGTCCTCAC3'. The oligonucleotides were designed using the software VectorNTI (Invitrogen, CA, USA), on the basis of the following GenBank sequences: HSV 1 strain 17 (NC_001806), HSV2 strain HG52 (NC_001798), B virus strain E2490 (NC_004812), SA8 (NC_006560), HVP2 (NC_007653), and contained HindIII and NdeI restriction sites at the 3' and 5' ends respectively, for cloning into the *E. coli *expression vector pET28a(+) (Novagen, CA, USA). The US11 clones, containing an amino terminal hexahistidine tag, were produced in BL21 (DE3) competent *E. coli *cells (Novagen, CA, USA) and sequenced for confirmation at the DNA Core Facility (National Microbiology Laboratory, Winnipeg).

For expression of the US11 proteins, *E. coli *clones were grown in 1L of LB medium to an OD_600 _of about 0.6 in the presence of 50 μg/ml of kanamycin. US11 was induced by adding 1 mM isopropyl-β-thiogalatopyranoside (IPTG, Novagen) and incubated at 25°C. Cells were harvested by centrifugation at 10 000 rpm for 10 minutes, resuspended in lysis buffer (20 mM Tris-HCL, 10 mM imidazole, 1% triton x-100, 10 mM β-mercaptoethanol, pH 8.0) with the addition of a protease inhibitor cocktail (Sigma, ON, Canada) and sonicated 5 times for 20 second intervals. Soluble and insoluble fractions of protein were separated by centrifugation at 14000 rpm. Most of the US11 was found in the soluble fractions and these fractions were stored at -20°C and used in the experiments described in this work.

### Purification of US11 Proteins

US11 proteins expressed with a His tag were purified on a Ni affinity column, prepared by equilibrating Ni resin (Profinity™ IMAC Ni-charged resin, Biorad, USA) with 1X binding buffer (20 mM Tris-HCL pH 7.4, 10 mM imidazole, 1 M NaCl, 1% triton x-100, 10 mM β-mercaptoethanol). *E. coli *extracts were mixed with an equal volume of 2X binding buffer (20 mM Tris-HCL pH 7.4, 10 mM imidazole, 2 M NaCl, 1% triton x-100, 10 mM β-mercaptoethanol) and applied to the affinity column. The addition of 1 M NaCl in the binding buffer was necessary to dissociate RNA that was bound to the US11 protein after lysis. The Ni column was washed with 10 bed volumes of wash buffer 1 (20 mM Tris-HCL, 10 mM imidazole, 1 M NaCl, 1% triton x-100, 10 mM β-mercaptoethanol pH 7.4) and then wash buffer 2 (20 mM Tris-HCL pH 7.4, 10 mM imidazole, 300 mM NaCl, 1% triton x-100, 10 mM β-mercaptoethanol). US11 was eluted with elution buffer (20 mM Tris-HCL pH 7.4, 300 mM imidazole, 300 mM NaCl, 1% triton x-100, 10 mM β-mercaptoethanol).

Ni column fractions containing US11 protein were pooled and loaded onto a 1 ml HiTrap heparin HP column (GE Healthcare) apparatus an AKTA FPLC system (GE Healthcare, QC, Canada), using phosphate buffer (10 mM NaPO_4 _pH 7.4). The US11 protein was eluted with a gradient of NaCl in phosphate buffer from 0 to 1.5 M, at a flow rate of 1 ml/min. Fractions containing US11 were confirmed by SDS-PAGE on a 15% SDS-PAGE gel and the protein concentration was measured using the RcDc Protein Assay kit (Biorad). The identity of US11 was confirmed by western blot analysis with an anti-polyhistidine mouse monoclonal antibody (Sigma), and by mass spectrometry at the Proteomics Core facility of the National Microbiology Laboratory, Winnipeg, Manitoba.

### Electrophoretic Mobility Shift Assays (EMSAs)

The RNA used for detecting protein binding was a randomly designed 42 bp double stranded synthetic fragment of sequence 5'UUCUCAAGUGAAGUCUGCUGAAGUACGUAACCUUAGAUACAU3' (Invitrogen). A dsDNA fragment of corresponding sequence was used as a binding control. Binding reactions were prepared using varying concentration of purified US11 protein (4 ng - 366 ng) and 20 ng of dsRNA in binding buffer (150 mM KCl, 0.1 mM DTT, 0.1 mM EDTA and 50 mM Tris, pH 7.4) in a final reaction volume of 20 μl. US11 and dsRNA were allowed to bind at room temperature for 30 minutes without agitation. An aliquot of gel loading buffer (0.25% bromophenol blue, 0.25% xylene cyanol, 15% ficoll-type 400) was added to the reaction mixtures and resolved on 10% non-denaturing polyacrylamide gels in 1X TBE.

### Filter Binding Assays

US11/dsRNA binding reaction was performed in 2 ml of binding buffer (150 mM KCl, 0.1 mM DTT, 0.1 mM EDTA and 50 mM Tris, pH 7.4) containing 5% Ni resin, a fixed amount of purified US11 protein and variable concentrations of ^32^P labeled dsRNA, obtained by serially diluting a solution of 31 nM of dsRNA trace-labeled with about 2 × 10^6 ^cpm of end-labeled RNA. With this procedure all the dilutions of dsRNA had the same specific activity. The tubes were incubated for 25 minutes at room temperature with gentle mixing and then loaded onto Whatman GF/C filters on a vacuum manifold (VWR). The reactions were filtered immediately and the filters were washed 3 times with 10 mL of ice cold 1X binding buffer. The radioactivity bound to the filter was counted using a LS 6500 scintillation counter.

Background radioactivity bound to the filter was determined by running in parallel for each dsRNA concentration controls containing all the reagents except for the US11 protein.

### Determining Kd Values and Statistical Analysis

A specific activity for each experiment was calculated as the average cpm/nmoles for each dsRNA concentration. The pmoles of dsRNA bound to the filter for each dsRNA concentration was calculated as:

$$\text{pmoles }=\frac{\text{cpm in sample }-\text{ cpm blank}}{\text{specific activity}}$$

The dissociation constant (Kd) and the total amount of binding sites [P] was calculated by non-linear regression of the equilibrium binding equation:

$$\left[\text{P}\cdot \text{RNA}\right]\;=\left[\text{P}\right]\left[\text{RNA}\right]∕\left(\text{Kd}+\left[\text{RNA}\right]\right)$$

Where: [P·RNA] is the amount of protein and RNA complexes as calculated from the binding experiments and [RNA] is the concentration of dsRNA in each point. The software also estimated the standard error of the calculated Kd and [P].

Several experiments were performed to determine the Kd of each US11 protein. A weighted average of the Kd values and standard deviation of the weighted average was calculated according to the formulas from Bevington and Robinson [21]. A one-way ANOVA test was performed to establish whether the Kd values were significantly different. GraphPad Prism^® ^software was used for these analyses.

## Results

### Protein expression and RNA binding activity

**Figure 1:**
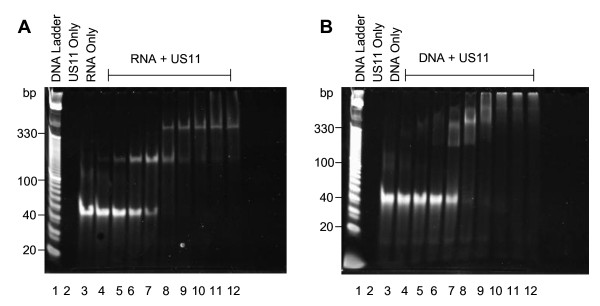
**EMSA of US11 protein from HSV1, to demonstrate its ability to bind to nucleic acids**. Increasing concentration of US11 protein ranging from 4.3-112 ng (from lane 4 to 12, respectively) were incubated with 20 ng of nucleic acid substrate and then run on a 10% acrylamide gel and stained with ethidium bromide. A) A 42 bp dsRNA was used as substrate. B) A 42 bp dsDNA with sequence corresponding to the dsRNA was used as substrate.

RNA-binding activity of the expressed and purified US11 proteins was assessed by electrophoretic mobility shift assay (EMSA). Figure 1A shows the binding activity of US11 from HSV 1 to the 42 base pair random sequence of RNA, as described in Materials and Methods. We chose the length of 42 base pairs based on previous work which showed that US11 from HSV1 binds a minimum of 39 to a maximum of 300 bp of dsRNA efficiently [12].

**Figure 2:**
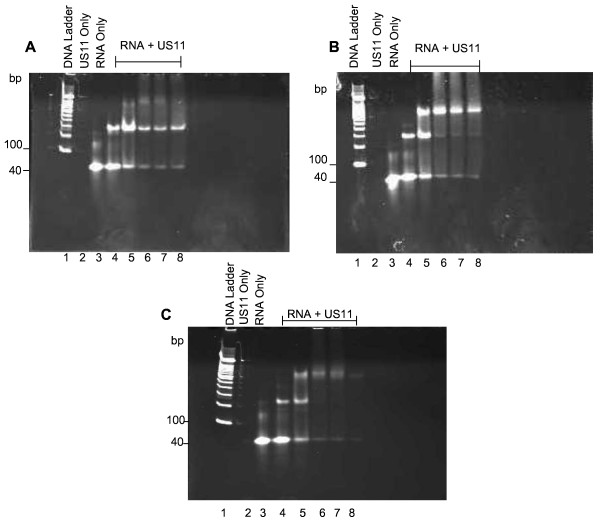
**EMSAs of US11 from HVP2 (A), HSV 2 (B) and B virus (C)**. This figure demonstrates the ability of US11 from the various viral sources to bind to RNA. Increasing concentrations of US11 protein were incubated with 20 ng of nucleic acid substrate and run on a 10% acrylamide gel stained with ethidium bromide. A) US11 from HVP2 is bound to RNA. Concentrations of protein include: 23 ng - 366 ng (lanes 4-8, respectively). B) US11 from HSV2 is bound to RNA. Concentrations of protein include: 22 ng - 354 ng (lanes 4-8, respectively). C) US11 from B virus is bound to RNA. Concentrations of protein include: 22 ng - 348 ng (lanes 4-8, respectively).

At lower concentrations of protein (lanes 4-7; 4.3 ng, 6.4 ng, 9.7 ng and 14.5 ng US11) only one shifted band is visible by ethidium bromide staining. A second band of higher molecular weight (MW) appears at higher concentrations of protein (lanes 8-10; 21.8 ng, 32.6 ng and 49.0 ng US11) and some RNA forms a high MW smear or remains trapped in the gel wells at the highest protein concentration (lanes 11-12; 73.5 and 110 ng US11). This result indicates that more than one molecule of US11 protein can bind to one molecule of dsRNA. All US11 proteins from HSV 1, HSV 2, B virus and HVP2 remained active post-purification as they all bound dsRNA with similar kinetics (Figure 2). US11 from SA8 did not express in the *E. coli *system and therefore US11 from SA8 was not used in the rest of the study.

US11 has been described predominantly as an RNA binding protein, although some previous reports in the literature show that US11 is also able to bind dsDNA [14,15]. Figure 1B shows that US11 does bind dsDNA but with a lower affinity and not in a discernable stoichiometric manner. The dsDNA substrate used for this experiment had a nucleotide sequence corresponding to the sequence of the dsRNA substrate.

### Determination of Kd for US11 proteins from HSV1, HSV2, B virus and HVP2

In order to determine the Kd of US11 proteins for dsRNA we used the filter binding assay described in detail in Materials and Methods. For this assay the purified His tagged US11 proteins are bound to Ni resin and, after incubation with 32P-labelled RNA substrate, the free radioactivity is removed by filtration. This classic binding method has the advantage over EMSA of the possibility of measuring the real equilibrium binding, by using excess amounts of RNA substrate and rapidity of washing, as opposed to the small sample volume for electrophoresis and the many hours of an electrophoresis run, during which a substantial portion of RNA bound protein may dissociate. In addition, filter binding is more quantitative than measuring radioactivity on X-ray image or a cut gel slice.

**Figure 3:**
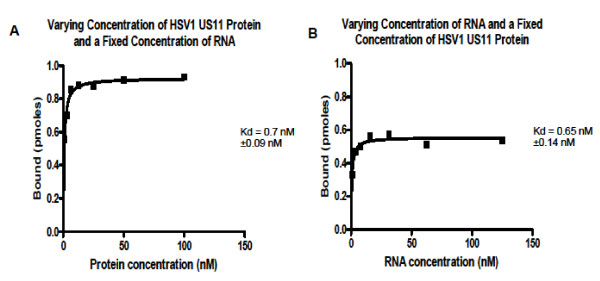
**Determination of Kd of binding to dsRNA of US11 protein from HSV-1**. The Kd was calculated by non-linear regression as described in Materials and methods and it is reported on the figure together with the estimated standard error. A) The Kd was determined with a fixed concentration of dsRNA (1 nM) and a range of concentration of proteins from 1 to 100 nM. B) The Kd was determined with a fixed concentration of protein (2 nM) and a range of concentrations of dsRNA from 0.97 to 125 nM.

In order to confirm that the filter binding method was free of artifacts that could influence the calculation of Kd, we performed two binding kinetic experiment, using a fixed concentration of RNA and varying concentrations of Us11 (Figure 3A), or using varying concentrations of RNA and a fixed concentration of Us11 protein from HSV1 (Figure 3B). The calculated Kd were 0.65 ± 0.14 nM and 0.70 ± 0.09 nM, and they were not significantly different as determined by an unpaired t-test (p value = 0.7), indicating that the true Kd value was determined.

In order to accurately measure and compare the Kd of the US11 proteins from HSV1, HSV2, HVP2 and B virus, we performed filter binding assays over a range of dsRNA concentrations of 31 nM - 200 nM in parallel for the four viruses.

Table 1 shows the results of 5 independent binding experiments. For each experiment the Kd was calculated by non-linear regression of the binding equation described in Materials and Methods (as done for example in Figure 3A) and the standard errors were also estimated. The last line of table 1 reports the weighted mean of the Kd for each US11 protein and the weighted standard deviation. The standard error of the Kd calculated by the non-linear regression was used as weight. None of the differences between Kd values for different US11 proteins were statistically significant, as determined by a one-way ANOVA test.

**Table 1:** Determination of the Kd of binding to dsRNA of US11 proteins from four simplexviruses.

Experiment	HSV1	HSV2	B virus	HVP2	p
1^a^	0.46 ± 0.40	0.29 ± 0.14	0.35 ± 0.28	0.42 ± 0.31	0.978
2 ^a^	2.34 ± 0.95	0.30 ± 0.52	6.07 ± 1.40	6.28 ± 3.09	0.068
3 ^a^	2.60 ± 1.13	1.14 ± 0.29	4.36 ± 1.35	6.10 ± 1.58	0.039
4 ^a^	1.84 ± 0.37	1.38 ± 0.15	1.69 ± 2.35	1.13 ± 0.27	0.975
5 ^a^	1.25 ± 0.26	1.66 ± 0.60	2.23 ± 0.94	2.58 ± 0.28	0.408

w. mean^b^	1.43 ± 0.29	0.89 ± 0.42	1.82 ± 3.42	1.80 ± 3.43	0.92

^a ^Kd calculated by non-linear regression ± estimated standard errors

^b ^weighted average calculated using Kd standard errors as weights ± weighted standard deviation

There was some variation in the Kd determinations among different experiments, but, except for experiment 3, Kds for different viruses were not significantly different also within each experiment (Table 1, rightmost column).

## Discussion

US11 inhibits interferon-mediated shutoff of protein synthesis by the PKR system, but its exact role during the course of herpesvirus infection still remains unclear. US11 is not essential for HSV replication in culture [22], but HSV also produces a second protein that inhibits PKR, γ_1_34.5, which is in turn essential for infectivity in cells and mice. In contrast, simian simplexviruses lack a γ_1_34.5 gene and US11 seems to be the only protein with PKR inhibitory activity. In order to investigate possible differences in activity between human and simian simplexviruses, we have studied the dsRNA binding activity of US11 expressed from HSV-1, HSV-2, HVP-2 and B virus.

Our results show that US11 proteins from HSV-1, HSV-2, HVP-2 and B virus bind the dsRNA substrate with the same affinity. In particular, there was no difference in Kd for US11 of HVP-2, despite the fact that this protein has a higher number of dsRNA-binding repeats. We determined the average Kd value for a synthetic dsRNA substrate at 1.07 ± 1.86 nM. Previous studies [12,20] have reported Kd values for US11 for different dsRNA species and showed that the affinity for dsRNA varied and was impacted by the length of the nucleic acid. Bryant *et al. *reports a Kd of 70 nM in the case of US11 for a 67 bp dsRNA fragment which was achieved by electrophoretic mobility-shift assays. They were also able to map the binding site within a 46 base pair segment of the RNA. On the other hand Khoo *et al. *reported an apparent Kd value of 12.6 nM for a 81 bp dsRNA species using a filter binding assay [12]. The Kd values obtained thus far for US11 from HSV1 may be dependent on the size of the dsRNA fragment and probably also on the techniques used to establish the Kd value.

Our Kd is considerably lower of the range reported in the literature, but the method we used is more accurate than gel retardation technique used in the previous literature. Filter binding technique allows to work at equilibrium binding and in the presence of excess ligand. Binding experiments under conditions of excess of dsRNA or excess of US11 produced similar Kd values (Figure 3), indicating that the filter binding assays truly measures equilibrium binding.

In conclusion, there is no difference in dsRNA binding activity among the US11 proteins of these four simplexviruses, also despite the higher number of repeats of the R-X-P RNA binding motif. However, Us11 may bind RNA as a means of homing to PKR and physically inhibit the enzyme, and studies have shown that US11 forms a physical association with PKR [23]. It is also clear that US11 inhibits PKR [11,15,16]. Therefore it is possible that difference in expression kinetics of Us11 or differences in the interaction of Us11 with PKR may account for the ability of simian simplexviruses to function without expressing the γ_1_34.5 protein. Future research should compare the effect of Us11 from different simplexvirusees on the phosphorylation levels of PKR or eIF2α, *in vitro *or in cells infected by human or simian simplexviruses.

## Competing interests

The authors declare that they have no competing interests.

## Authors' contributions

ST expressed and purified the US11 proteins, performed the binding experiments and analyzed the results. CDC developed the methodology for cloning and purification of the US11 proteins. AS designed and supervised this project and contributed to the analysis of the data. All authors contributed in the writing and final approval of this manuscript.

## References

[B1] Rogers KM Ealey KA Ritchey JW Black DH Eberle R Pathogenicity of different baboon herpesvirus papio 2 isolates is characterized by either extreme neurovirulence or complete apathogenicity J Virol 2003 77 10731 10739 10.1128/JVI.77.20.10731-10739.2003 14512523 PMC224954

[B2] Ritchey JW Ealey KA Payton ME Eberle R Comparative pathology of infections with baboon and African green monkey alpha-herpesviruses in mice J Comp Pathol 2002 127 150 161 10.1053/jcpa.2002.0575 12354526

[B3] Perelygina L Zhu L Zurkuhlen H Mills R Borodovsky M Hilliard JK Complete Sequence and Comparative Analysis of the Genome of Herpes B Virus (Cercopithecine Herpesvirus 1) from a Rhesus Monkey J Virol 2003 77 6167 6177 10.1128/JVI.77.11.6167-6177.2003 12743273 PMC155011

[B4] Tyler SD Peters GA Severini A Complete genome sequence of cercopithecine herpesvirus 2 (SA8) and comparison with other simplexviruses Virology 2005 331 429 440 10.1016/j.virol.2004.09.042 15629785

[B5] Tyler SD Severini A The complete genome sequence of herpesvirus papio 2 (Cercopithecine herpesvirus 16) shows evidence of recombination events among various progenitor herpesviruses J Virol 2006 80 1214 1221 10.1128/JVI.80.3.1214-1221.2006 16414998 PMC1346941

[B6] Pasieka TJ Baas T Carter VS Proll SC Katze MG Leib DA Functional Genomic Analysis of Herpes Simplex Virus Type 1 Counteraction of the Host Innate Response J Virol 2006 80 7600 7612 10.1128/JVI.00333-06 16840339 PMC1563739

[B7] Chou J Roizman B The gamma 1(34.5) gene of herpes simplex virus 1 precludes neuroblastoma cells from triggering total shutoff of protein synthesis characteristic of programed cell death in neuronal cells Proc Natl Acad Sci USA 1992 89 3266 3270 10.1073/pnas.89.8.3266 1314384 PMC48847

[B8] Cassady KA Gross M Roizman B The herpes simplex virus US11 protein effectively compensates for the gamma1(34.5) gene if present before activation of protein kinase R by precluding its phosphorylation and that of the alpha subunit of eukaryotic translation initiation factor 2 J Virol 1998 72 8620 8626 9765401 10.1128/jvi.72.11.8620-8626.1998 PMC110273

[B9] Mohr I Gluzman Y A herpesvirus genetic element which affects translation in the absence of the viral GADD34 function EMBO J 1996 15 4759 4766 8887567 PMC452208

[B10] Mulvey M Poppers J Ladd A Mohr I A herpesvirus ribosome-associated, RNA-binding protein confers a growth advantage upon mutants deficient in a GADD34-related function J Virol 1999 73 3375 3385 10074192 10.1128/jvi.73.4.3375-3385.1999 PMC104102

[B11] Poppers J Mulvey M Khoo D Mohr I Inhibition of PKR activation by the proline-rich RNA binding domain of the herpes simplex virus type 1 Us11 protein J Virol 2000 74 11215 11221 10.1128/JVI.74.23.11215-11221.2000 11070019 PMC113216

[B12] Khoo D Perez C Mohr I Characterization of RNA determinants recognized by the arginine- and proline-rich region of Us11, a herpes simplex virus type 1-encoded double-stranded RNA binding protein that prevents PKR activation J Virol 2002 76 11971 11981 10.1128/JVI.76.23.11971-11981.2002 12414939 PMC136894

[B13] Benboudjema L Mulvey M Gao Y Pimplikar SW Mohr I Association of the herpes simplex virus type 1 Us11 gene product with the cellular kinesin light-chain-related protein PAT1 results in the redistribution of both polypeptides J Virol 2003 77 9192 9203 10.1128/JVI.77.17.9192-9203.2003 12915535 PMC187382

[B14] Roller RJ Monk LL Stuart D Roizman B Structure and function in the herpes simplex virus 1 RNA-binding protein U(s)11: mapping of the domain required for ribosomal and nucleolar association and RNA binding in vitro J Virol 1996 70 2842 2851 8627758 10.1128/jvi.70.5.2842-2851.1996 PMC190141

[B15] MacLean CA Rixon FJ Marsden HS The products of gene US11 of herpes simplex virus type 1 are DNA-binding and localize to the nucleoli of infected cells J Gen Virol 1987 68 1921 1937 10.1099/0022-1317-68-7-1921 3037015

[B16] Mulvey M Camarena V Mohr I Full resistance of herpes simplex virus type 1-infected primary human cells to alpha interferon requires both the Us11 and gamma(1)34.5 gene products J Virol 2004 78 10193 10196 10.1128/JVI.78.18.10193-10196.2004 15331752 PMC514974

[B17] Mulvey M Poppers J Sternberg D Mohr I Regulation of eIF2alpha phosphorylation by different functions that act during discrete phases in the herpes simplex virus type 1 life cycle J Virol 2003 77 10917 10928 10.1128/JVI.77.20.10917-10928.2003 14512542 PMC225003

[B18] Peters GA Khoo D Mohr I Sen GC Inhibition of PACT-mediated activation of PKR by the herpes simplex virus type 1 Us11 protein J Virol 2002 76 11054 11064 10.1128/JVI.76.21.11054-11064.2002 12368348 PMC136652

[B19] Sanchez R Mohr I Inhibition of cellular 2'-5' oligoadenylate synthetase by the herpes simplex virus type 1 Us11 protein J Virol 2007 81 3455 3464 10.1128/JVI.02520-06 17229694 PMC1866071

[B20] Bryant KF Cox JC Wang H Hogle JM Ellington AD Coen DM Binding of herpes simplex virus-1 US11 to specific RNA sequences Nucleic Acids Res 2005 33 6090 6100 10.1093/nar/gki919 16246910 PMC1266072

[B21] Bevington PR Robinson DK Data Reduction and Error Analysis for the Physical Sciences 2003 McGraw-Hill, New York

[B22] Brown SM Harland J Three mutants of herpes simplex virus type 2: one lacking the genes US10, US11 and US12 and two in which Rs has been extended by 6 kb to 0.91 map units with loss of Us sequences between 0.94 and the Us/TRs junction J Gen Virol 1987 68 1 18 10.1099/0022-1317-68-1-1 3027237

[B23] Cassady KA Gross M The herpes simplex virus type 1 U(S)11 protein interacts with protein kinase R in infected cells and requires a 30-amino-acid sequence adjacent to a kinase substrate domain J Virol 2002 76 2029 2035 10.1128/jvi.76.5.2029-2035.2002 11836380 PMC135940

